# Development of Threat Expression Following Infant Maltreatment: Infant and Adult Enhancement but Adolescent Attenuation

**DOI:** 10.3389/fnbeh.2019.00130

**Published:** 2019-06-25

**Authors:** Anouchka Junod, Maya Opendak, Joseph E. LeDoux, Regina M. Sullivan

**Affiliations:** ^1^Emotional Brain Institute, Nathan Kline Institute, Orangeburg, NY, United States; ^2^Child and Adolescent Psychiatry, New York University School of Medicine, New York, NY, United States; ^3^Center for Neural Science, New York University, New York, NY, United States

**Keywords:** infant, trauma, maltreatment, learned fear, expression threat, amygdala, medial prefrontal cortex

## Abstract

Early life maltreatment by the caregiver constitutes a major risk factor for the development of later-life psychopathologies, including fear-related pathologies. Here, we used an animal model of early life maltreatment induced by the Scarcity-Adversity Model of low bedding (LB) where the mother is given insufficient bedding for nest building while rat pups were postnatal days (PN) 8–12. To assess effects of maltreatment on the expression of threat-elicited defensive behaviors, animals underwent odor-shock threat conditioning at three developmental stages: late infancy (PN18), adolescence (PN45) or adulthood (>PN75) and tested the next day with odor only presentations (cue test). Results showed that in typically developing rats, the response to threat increases with maturation, although experience with maltreatment in early infancy produced enhanced responding to threat in infancy and adulthood, but a decrease in maltreated adolescents. To better understand the unique features of this decreased threat responding in adolescence, c-Fos expression was assessed within the amygdala and ventromedial prefrontal cortex (vmPFC) associated with the cued expression of threat learning. Fos counts across amygdala subregions were lower in LB rats compared to controls, while enhanced c-Fos expression was observed in the vmPFC prelimbic cortex (PL). Correlational analysis between freezing behavior and Fos revealed freezing levels were correlated with CeA in controls, although more global correlations were detected in LB-reared rats, including the BA, LA, and CeA. Functional connectivity analysis between brain regions showed that LB reared rats exhibited more diffuse interconnectivity across amygdala subnuclei, compared the more heterogeneous patterns observed in controls. In addition, functional connectivity between the IL and LA switched from positive to negative in abused adolescents. Overall, these results suggest that in adolescence, the unique developmental decrease in fear expression following trauma is associated with distinct changes in regional function and long-range connectivity, reminiscent of pathological brain function. These results suggest that early life maltreatment from the caregiver perturbs the developmental trajectory of threat-elicited behavior. Indeed, it is possible that this form of trauma, where the infant’s safety signal or “safe haven” (the caregiver) is actually the source of the threat, produces distinct outcomes across development.

## Introduction

In altricial species, such as humans and rodents, the brain continues to develop after birth and is quite sensitive to environmental programming that permits adaptation to diverse environments and cultures. However, this open system also leaves the brain vulnerable to programming by trauma, with programming that goes beyond adaptation to initiate a pathological developmental pathway. Childhood trauma experiences, especially when associated with the caregiver (as occurs in maltreatment), are associated with mental health issues, including PTSD, anxiety and other threat processing-related pathologies (De Bellis et al., 1999; Anda et al., 2006; Andersen et al., 2008; McEwen and Gregerson, 2019; Van Assche et al., 2019), which has been modeled in rodents and non-human primates (Harlow and Harlow, 1965; Brenhouse and Andersen, 2011; Drury et al., 2015; Perry et al., 2017; Callaghan et al., 2019). One challenge to understanding how maltreatment causes threat-associated pathologies is the protracted and dynamic maturation of the threat system, which morphs through childhood, adolescence and adulthood as life and environmental demands change to produce unique expression patterns across the lifespan (Anda et al., 2006; Moffitt et al., 2007; Gilbert et al., 2009; Green et al., 2010; Callaghan et al., 2019). Here, using a rat animal model, we assessed how responses to a learned threat (following threat conditioning) changes as pups begin the transition from dependence on the mother [postnatal day (PN) 18] to adolescence, and finally adulthood, following typical maternal care and early life maltreatment. To model maltreatment, we employ the Scarcity-Adversity Model of low bedding (LB, insufficient bedding for nest building to induce maltreatment of pups) from PN8-12 (Opendak and Sullivan, 2016; Walker et al., 2017; Perry et al., 2018; Watamura and Roth, 2018).

Exploring how trauma contributes to the pathogenesis of anxiety and other threat-related disorders relies on understanding the typically developing threat response system and how it morphs as the developing organism matures and changes ecological niches. The infant system is not an immature version of the adult threat system (Landers and Sullivan, 2012). Specifically, within the immature threat system, the young seek and approach the caregiver when threatened and the caregiver responds to the threat while protecting the infants. This is seen in children as they approach the attachment figure (i.e., parent, guardian) or “safe base” for protection when threatened (Kerns et al., 2015). The developmental transition to mature self-defense occurs gradually during maturation, with infants approaching independence sometimes engaging in defensive behaviors such as freezing, or more active responses such as escape or fleeing, as these skills develop and the infant is more frequently away from the caregiver (Callaghan et al., 2019). It is this age range in which we begin our neurobehavioral analysis in infant (PN18) rats. With further physical and cognitive maturation, pups leave the mother and responding to threat is completely independent: here, we explore adolescent (PN45) and adult responses to provide a lifespan assessment. This complements research from other labs, as well as our own, studying threat conditioning across development (Pattwell et al., 2011; Li et al., 2012; Cowan et al., 2013; Poulos et al., 2014; Hartley and Lee, 2015; Madsen and Kim, 2016; Bucci and Stanton, 2017; Tallot et al., 2017; Davis et al., 2018).

Animal models have shown that developmental behavioral changes are reflected in the dynamic changes in the neural substrates, including developmental changes in responding to threats. Specifically, the functional emergence of learned and innate threat-elicited behavior at PN10 (Sullivan et al., 2000; Wiedenmayer and Barr, 2001; Moriceau et al., 2004; Takahashi, 2014; Santiago et al., 2018) is dependent upon the developmental emergence of the amygdala (Sullivan et al., 2000). Through PN15, amygdala-dependent learning is suppressed if the mother is present (Moriceau et al., 2006; Upton and Sullivan, 2010). In rat pups PN16 and older, amygdala-dependent cue learning and expression occurs with or without the mother present. Similarly to younger pups, there is suppression of learning about threat if the mother is present, although adults and not pups use the PFC to reduce acquisition (Levine, 2001; Inagaki and Eisenberger, 2012; Hornstein et al., 2016; Opendak et al., 2019; Robinson-Drummer et al., 2019). In particular, the PFC subareas corresponding to the infralimbic (IL) and prelimbic (PL) cortices (Brodmann areas 25 and 32, respectively) have been shown to modulate amygdala in the expression and suppression of learned threat (Linnman et al., 2012; Motzkin et al., 2015; Corches et al., 2019). However, while basic features of threat conditioning appear similar over much of development, the neural substrates and key features of threat conditioning and its expression differ in infants and adults: both the PFC and hippocampus and supporting behaviors such as extinction and context learning functionally emerge around to the age of weaning, at around PN23 (Rudy and Morledge, 1994; Barnet and Hunt, 2005; Yap and Richardson, 2005; Kim et al., 2009; Raineki et al., 2010a; Chan et al., 2011; Callaghan and Richardson, 2012; Li et al., 2012; Poulos et al., 2014). We also focus on adolescence, a period of increased prevalence of psychopathology involving perturbation of emotion (Giedd et al., 1999; Spear, 2000; Teicher et al., 2003; Andersen and Teicher, 2008; Monk et al., 2008; Raineki et al., 2012; Pattwell et al., 2016). Furthermore, work in peri-adolescent rodents has uncovered unique behavioral patterns of threat learning and memory extinction, processes which have shown sensitivity to developmental events (Stanton et al., 1987; Kim et al., 2009; Pattwell et al., 2011; Callaghan and Richardson, 2012; Robinson-Drummer and Stanton, 2015; Pattwell and Bath, 2017; Bath, 2018). Here, we take a lifespan approach and question how the behavioral expression to a learned threat changes as the infant transitions across infancy, adolescence and adulthood. In particular, we explore how threat responses such as freezing, as well as more active defensive responses such as fleeing, changes across development.

Experiencing infant trauma and maltreatment from the caregiver has major effects on threat detection, as reflected in defensive behavior, and, on key structures within the defensive network, such as the amygdala and PFC, in both humans and animal throughout development (De Bellis et al., 1999; Gunnar et al., 2007; Lupien et al., 2009; Dannlowski et al., 2013; Gee et al., 2013b; Birn et al., 2014; Opendak and Gould, 2015; Fonzo et al., 2016; Opendak and Sullivan, 2016; Teicher et al., 2016; Heany et al., 2018; Lange et al., 2018; Roquet et al., 2018; Santiago et al., 2018; Watamura and Roth, 2018; Callaghan et al., 2019). Less well understood is how the trauma effects change during development. The effects of maltreatment can be seen in childhood but identifying these subtle effects can be challenging until early adolescence when psychiatric disorders, as well as fear pathologies, increasingly emerge, with the amygdala and PFC targeted (Demers et al., 2018; Roos et al., 2018; Torrisi et al., 2018). The protracted development of the brain likely contributes to these developmental transitions (Giedd et al., 1999; Lupien et al., 2009; Raineki et al., 2010a,b; Rincon-Cortes et al., 2015; Teicher et al., 2016; Opendak et al., 2017; Hagler et al., 2018; Heany et al., 2018; Hodel, 2018; Lange et al., 2018; VanTieghem and Tottenham, 2018; Botros et al., 2019).

Here, we use the rodent Scarcity-Adversity Model of low bedding (LB) to induce infant maltreatment by the mother by providing insufficient bedding for nest building. We target this treatment to PN8-12 in the rodent pup; though there is no consensus mapping rodent age onto human age, this developmental period corresponds roughly to toddlerhood (Callaghan et al., 2019). We use learning about threat (Pavlovian threat conditioning) following perturbed development using LB maltreatment or typical control rearing to explore transitions in threat expression. We focus on two brain areas critical for responses to threat, the amygdala and ventromedial prefrontal cortex (vmPFC), during adolescence to complement the existing literature on fear/threat development, amygdala and PFC function in infancy and adulthood (Rudy and Morledge, 1994; Fanselow and LeDoux, 1999; Sevelinges et al., 2007; Thompson et al., 2008; Moriceau et al., 2009a,b; Cowan et al., 2013; Marek et al., 2013; Maren et al., 2013; Spielberg et al., 2014; Takahashi, 2014; Almada et al., 2015; Do-Monte et al., 2015; Duits et al., 2015; Pattwell et al., 2016; Matsuda et al., 2018; Norrholm and Jovanovic, 2018).

Our specific goals were to chart the neurobehavioral expression of threat learning across pre-weaning age (PN18), adolescence (PN45) and adulthood in typical and perturbed development. We hypothesized that, due to the unique neural circuitry supporting threat learning across development, the effects of maltreatment would be distinct at each stage of development.

## Materials and Methods

### Subjects

**FIGURE 1:**
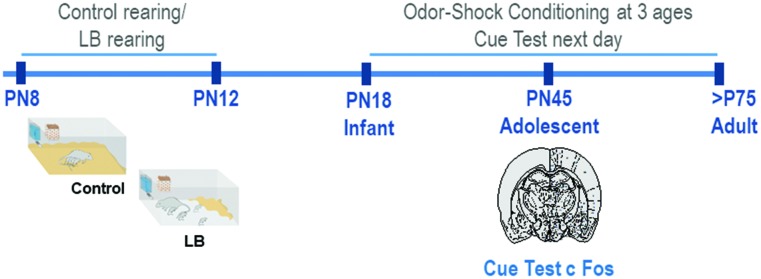
Schematic of methodology and experimental timeline. From PN8-12, pups were exposed to either Scarcity-Adversity Model of low bedding (LB) rearing or control rearing from the mother. For LB rearing, the mother was provided with insufficient bedding for nest building, which resulted in maltreatment of pups but growth similar to controls. Pups are odor-shock conditioned at one age, either during infancy (PN18) adolescence (PN45) or adulthood (>PN75). All animals were tested in a cue test the next day; in adolescents, neural responses following cue testing were assessed with c-Fos.

Male and female Long-Evans rat pups were bred in the Institute’s animal care facilities and housed (polypropylene cages 34 × 29 × 17 cm, wood chips, *ad libitum* food and water) in a temperature (20°C) and light (06:00–18:00 h) controlled room. The birthdate was PN0. Litters were culled to 12 pups on PN1 and only 1 male and 1 female per litter was used in any conditioning/test condition. For pre-weaning analyses (PN18) males and females were used; sex differences in the response to threat have not been shown at this age (Robinson-Drummer et al., 2019 under revision). For analyses at PN45 and adulthood, only males were used (see Figure 1 for Experimental Timeline). Procedures were approved by the Institutional Animal Care and Use Committee and followed National Institutes of Health guidelines.

### Scarcity-Adversity Model of Low Bedding (LB)

**FIGURE 2:**
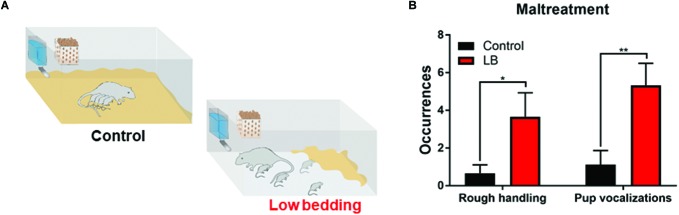
Low bedding induces maternal maltreatment of pups. **(A)** Pups were exposed to either Scarcity-Adversity Model of low bedding (LB) rearing or control rearing from postnatal days (PN)8-12. LB rearing involved providing the mother with insufficient bedding for nest building, which produces maltreatment of pups and increased pup vocalizations **(B)** but growth indistinguishable from controls. Maltreatment was verified by repeated observations of maternal behaviors categorized as rough handling, such as stepping on pups, transporting pups by a body area other than the nape of the neck (i.e., leg), or roughly moving pups within the nest.

We used a naturalistic stressed mother paradigm to induce maltreatment of pups over 5 days (from PN8 to PN12) by providing the mother with insufficient bedding: 1.3 cm layer compared to 0–0.5 cm layer covering the bottom of the cage (Roth and Sullivan, 2005; Walker et al., 2017). This limited bedding environment decreased the mother’s ability to construct a nest, which resulted in frequent nest building, and transporting/rough handling of pups (see Figure 2). Twice daily 15 min observation periods were undertaken on all 5 days of the manipulations to verify maltreatment in LB mothers and not in controls.

### Odor-Shock Conditioning

Learning was induced using a classical Pavlovian odor-shock conditioning protocol using a standard conditioning apparatus (Coulbourn Instruments, Whitehall, PA) (LeDoux et al., 1988; Fanselow and LeDoux, 1999; Maren, 2001; Johansen et al., 2011). Although this protocol is typically known as “fear” conditioning, we refer to this as threat conditioning to avoid the implication that a subjective state of fear is the result of conditioning (LeDoux, 2014). 8–10 animals were used per rearing condition/learning condition and each animal was only used at one age. Adult and adolescent animals were acclimated to the conditioning apparatus for 5–10 min. Un-weaned pups (i.e., infant PN18) were not handled so as not to disturb mother-infant interaction and potentially inadvertently stress pups. On the conditioning day, a 30s consecutive odor (peppermint) presentation co-terminated with a 1s shock. The conditioned stimulus (CS) odor was delivered by an olfactometer (2 L/min flow rate at a concentration of 1:10). The unconditioned stimulus (US) foot shock was delivered by a grid at an age-specific intensity (PN18: 0.5 mA; adolescent: 0.55–6 mA; adult: 0.65–0.7 mA). CS-US pairings had a 4–5 min intertrial interval (ITI). Three training conditions were used: paired (as noted above), unpaired and odor only (Sevelinges et al., 2008; Tallot et al., 2016; Opendak et al., 2018).

Conditioning occurred in chambers constructed of aluminum and Plexiglas with metal stainless steel rod flooring attached to a shock generator (Wilensky et al., 2006). Each chamber had a top-mounted camera for videotaping and later analysis of the behaviors of individual rats during training and was enclosed within a sound-isolated, and ventilated cubicle to ensure odor withdrawal.

The cue test, consisting of three 30 s odor presentations (same parameters as conditioning), was given 24 h after conditioning in a novel context (attenuation chamber, Med Associates VT) in a beaker (PN18 glass beaker 2000 ml, 4″d; adolescent glass beaker 4000 ml, 6″d; adult plastic 8500 ml, 8.5″d). We measured time spent freezing and brains were removed 90 min after this test.

### c-Fos Immunohistochemistry

As a global measure of neural activity, we assessed regional expression of the immediate early gene c-Fos, a proxy for neuronal activation. Brains were harvested 90 min after the cue test to assess the role of the amygdala, PL and IL during behavioral expression during a threat presentation using c-Fos (5–6 brains per condition were analyzed). Animals were decapitated, brains were removed within 3 min, frozen in isopentane and stored at -70°C until further processing using standard techniques (Sevelinges et al., 2007; Leuner et al., 2012). In the regions of interest (ROI), every 3rd coronal section (20 μm) was collected on pretreated slides (Fisherbrand Plus). Slices were post-fixed with 4% paraformaldehyde/100 mM phosphate buffered saline (PBS) and rinsed twice in 50 mM tris-HCl and 150 mM NaCl (TBS), and twice in TBS containing 0.05% Tween 20 (TBST). To eliminate endogenous peroxidase activity, sections were incubated in 70% Methanol, containing 1% hydrogen peroxide (H_2_O_2_). Following TBS and TBST rinse, unspecific epitopes of the brain slices were blocked by incubation in 2% Normal goat serum (Jackson ImmunoResearch) and 3% bovine serum albumin (Sigma Aldrich) in TBST for 2 h. Slides were further incubated overnight at 4°C in 2% Normal goat serum, 3% bovine serum albumin and primary c-Fos antibody (EMD Biosciences) diluted 1:5000 in TBST. Next they were rinsed in TBS and TBST and further incubated with a biotinylated secondary antibody (Jackson ImmunoResearch) for 2 h at room temperature and then incubated for 90 min in avidin-biotin-peroxidase (ABC) complex solution (Vectastain). Following washing in TBST, slides were treated with 2% 3,3′-diaminobenzidine and 0.06% H_2_O_2_ for 20 min. Brain slices were dehydrated in 70%, 95% and 100% ethanol solutions and Xylene (Thermo Fisher Scientific) and cover-slipped. Image acquisition was done at 10× using an Olympus BX51 microscope and the Neurolucida software (MBF Bioscience). c-Fos positive cells were counted using Image J software (NIH). Amygdala nuclei were outlined using a stereotactic atlas (Paxinos and Watson, 2013) and all c-Fos positive cells were counted bilaterally blind to treatment conditions. c-Fos positive cells were distinguished from the background by the density of staining, the shape and the size of the cell. The mean number of c-Fos positive cells per amygdala and prefrontal cortex nuclei for each animal was determined by averaging the total counts (both hemispheres) from three different sections.

### Functional Connectivity Analysis

For functional connectivity modeling, bivariate correlation matrices were created by computing ratios of mean c-Fos uptake for all pairwise combinations of brain regions. For quantitative analyses, each group’s correlation matrices were transformed into *z*-scores using the Fisher transformation and group differences between modules were analyzed by ANOVA and Bonferroni-corrected pairwise tests.

### Statistical Analysis

For behavioral conditioning data, 2-way repeated measured ANOVA was computed using CS-US pairing (three pairings) and rearing (LB vs. Control). Testing data was analyzed with 2-way ANOVA, comparing learning condition (paired, unpaired, odor only) and rearing (LB vs. Control). Fos data was analyzed in adolescents using 2-way ANOVA, comparing amygdala/vmPFC subnuclei and rearing condition; amygdala Fos and vmPFC Fos were analyzed in separate ANOVAs. To compare correlations, Pearson *r* values were converted into z-scores using the Fisher transformation.

## Results

### Scarcity-Adversity Model of Low Bedding (LB) Induced Maltreatment From PN8-12

During the bi-daily 15 min observation periods, maternal behaviors were scored and maltreatment verified (Raineki et al., 2010b). As illustrated in Figure 2, LB mothers show significantly more time stepping on and roughly handling pups and pups vocalized more (two-way ANOVA (rearing condition × behavior), ME of rearing [*F*_(1,31)_ = 13.58, *p* = 0.001]). Weight was continuously monitored at the different ages studied through development and no differences between groups were observed, consistent with previous work: at weanling, average weight of controls was 62.71 ± 2.31 g (*n* = 14) and LB average weight was 57.88 ± 2.94 g (*n* = 16); at PN45, average weight of controls was 196.8 ± 4.72 g (*n* = 36) and LB average weight was 198.8 ± 3.74 g (*n* = 40); at adulthood, average weight for controls was 371.3 ± 4.19 g (*n* = 14) and average weight for LB pups was 366.7 ± 8.08 g (*n* = 14).

### Odor-Shock Conditioning Across Development

**FIGURE 3:**
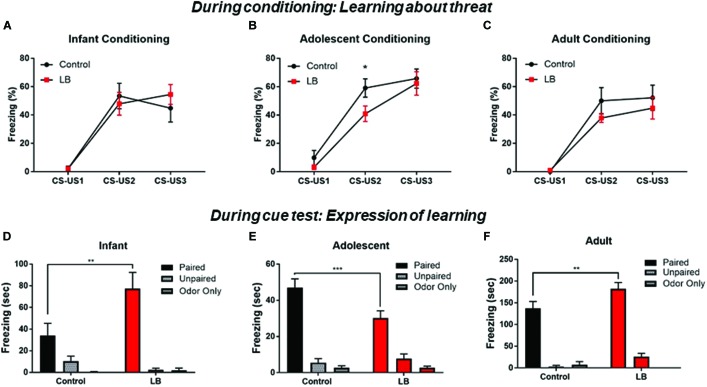
Acquisition and expression of learned threat across development. During conditioning, responses to the CS increased over consecutive CS-US pairings, a pattern indicative of learning. No statistical difference was seen between LB and control rearing as suggested by rate of learning for infants and adults **(A,C)**, although maltreated adolescents showed some retardation of learning **(B)**. Cue testing 24 h later in a novel environment was analyzed for percent time spent freezing to the CS at the three ages. All paired animals (control and LB) were significantly higher than unpaired and odor-only controls, although freezing to cues following maltreatment increased in infancy **(D)**, decreased in adolescence **(E)** and increased in adults **(F)**, relative to control-reared animals. ^∗∗^*p* < 0.01, ^∗∗∗^*p* < 0.001.

Overall, we observed that pups across development showed increased freezing to the paired CS-US cues over the course of training (Figure 3A–C). This was observed across ages and conditions [Infant: ME of cue presentation (*F*_(2,32)_ = 44.7, *p* < 0.000); Adolescent: ME of cue presentation (*F*_(2,56)_ = 71.17, *p* < 0.000); Adult: ME of cue presentation (*F*_(2,26)_ = 35.36, *p* < 0.000)]. However, previous maltreatment impaired acquisition at adolescence (Figure 3B), with decreased freezing during conditioning in the abused group [*t*_(84)_ = 2.151, *p* = 0.032].

### Expression of Learned Threat Throughout Development

CS cue testing was done 24 h after conditioning in a different context (3 CSs, odor and ITI same as conditioning). Overall, all paired animals at all ages and in both rearing conditions showed increased freezing to the CS relative to controls, indicating a learned association between the odor and the shock (Figure 3D–F).

For infants, an ANOVA revealed a significant interaction [*F*_(2,41)_ = 5.76; *p* < 0.006], no main effect of rearing condition [*F*_(1,41)_ = 3.449; *p* = 0.07] and a main effect of conditioning [*F*_(2,41)_ = 27.72; *p* = 0.0001]. *Post hoc* tests revealed that the Paired groups (LB and control) were always each significantly higher than unpaired and odor only groups (all *p*’s < 0.05). Freezing was higher in LB reared pups than controls in the paired conditioning groups [*t*_(41)_ = 3.82, *p* = 0.004].

For adolescents, an ANOVA revealed a significant interaction [*F*_(2,52)_ = 4.47; *p* = 0.016], no main effect of rearing [*F*_(1,52)_ = 2.582; *p* = 0.11] and a main effect of conditioning [*F*_(2,52)_ = 97.36; *p* < 0.001]. *Post hoc* tests revealed that the Paired groups (LB and control) were always each significantly higher than unpaired and odor only groups (all *p*’s < 0.05). Freezing levels were lower in LB reared pups than control-reared pups trained in the paired condition [*t*_(52)_ = 3.53, *p* < 0.001].

For adults, an ANOVA revealed no significant interaction [*F*_(2,31)_ = 2.307; *p* < 0.1164], no main effect of rearing [*F*_(1,31)_ = 3.925; *p* = 0.056] and a main effect of conditioning [*F*_(2,31)_ = 112.7; *p* = 0.0001]. *Post hoc* tests revealed that the Paired groups (LB and control) were each always significantly higher than unpaired and odor only groups (all *p*’s < 0.05). Freezing levels were higher in the LB pups compared to controls in the Paired groups [*t*_(31)_ = 2.976, *p* = 0.005].

### Amygdala Subnuclei and vmPFC IL and PL Cue Test Activation During Adolescence

**FIGURE 4:**
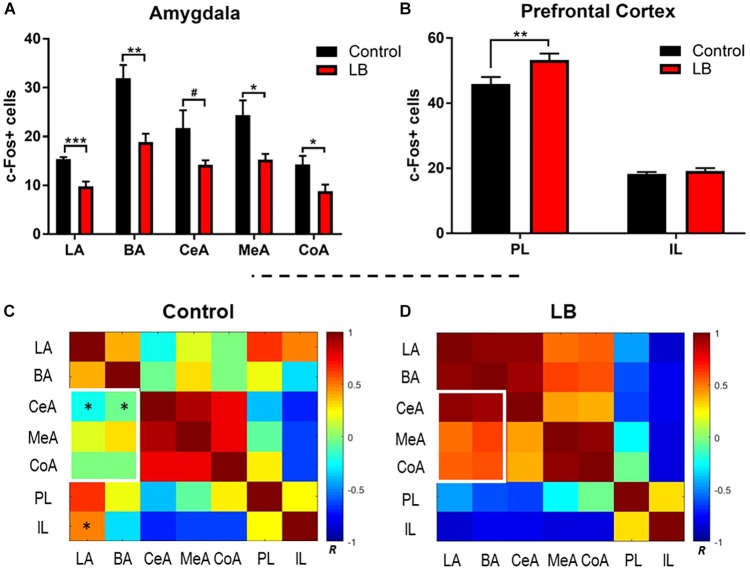
Amygdala and prefrontal cortex activation following memory retrieval at 24 h in adolescent animals. **(A)** Amygdala subnuclei activation following fear retrieval at 24 h in both control (black) and abused (red) animals; LA: lateral nucleus, B: basal nucleus, CeA: central nucleus, CoA: cortical nucleus, and MeA: medial nucleus. **(B)** Prefrontal cortex activation following fear memory retrieval at 24 h in both normal (black) and abused (red) animals; PL: prelimbic cortex, IL: infralimibic cortex. **(C,D)** Bivariate correlation matrices were contructed across regions assessed for c-Fos levels after cue test during adolescents following control or low bedding rearing. White squares denote example nodules (groups of regions) that show dramatic changes in connectivity across condition. Color bar shows Pearson’s *r* values with positive correlations in red and negative correlations in blue. For statistical comparisons, *r* values were converted to *z* scores via Fisher transform and compared using ANOVA. Asterisks indicate pairwise correlations that significantly differed between LB and control. ^∗^*p* < 0.05, ^∗∗^*p* < 0.01, ^∗∗∗^*p* < 0.001, and ^#^*p* = 0.05.

Our behavioral data indicate that adolescence represents an inflection point in the effects of maltreatment on the response to threat. To examine the neural substrates of this adolescent decrease in the fear response following maltreatment, c-Fos-positive cells were counted bilaterally in ROI, including the lateral, basal, central, medial and cortical nuclei of the amygdala, as well as the IL and PL of the prefrontal cortex, due to an established role for the vmPFC in the fear circuit (Figure 4). Laterality effects were not observed [three-way ANOVA (rearing × region × hemisphere), amygdala: no ME of hemisphere (*F*_(1,80)_ = 0.621, *p* = 0.433) or significant interactions; PFC: no ME of hemisphere (*F*_(1,40)_ = 0.821, *p* = 0.37)] and counts were therefore collapsed across hemispheres for further analysis. Within the amygdala, counts were analyzed via two-way ANOVA (rearing × region), which revealed main effects of both factors [region: *F*_(4,40)_ = 15.51, *p* < 0.0001; rearing: *F*_(1,40)_ = 40.36, *p* < 0.0001]. In accordance with the behavioral observations showing reduced freezing to the paired CS-US cue, amygdala subnuclei showed reduced activation in abused animals [Figure 4A; LA: *t*_(40)_ = 1.941, *p* = 0.059; BA: *t*_(40)_ = 4.568, *p* < 0.001; CeA: *t*_(40)_ = 2.634, *p* = 0.012; MeA: *t*_(40)_ = 3.157, *p* = 0.003; CoA: *t*_(40)_ = 1.909, *p* = 0.064].

We observed that early life abuse had a significant effect on the PL but not the IL (Figure 4B). Two way ANOVA (rearing × subregion) revealed an interaction [*F*_(1,20)_ = 4.434, *p* = 0.0481] as well as main effects of subregion [*F*_(1,20)_ = 401.3, *p* < 0.0001] and rearing [*F*_(1,20)_ = 7.191, *p* = 0.0143]. *Post hoc* tests revealed that c-Fos counts within the PL were significantly higher in the abused pups [*t*_(20)_ = 3.385, *p* = 0.002].

### Functional Connectivity Within the Amygdala-Prefrontal Network

Next, we compared how activity between subnuclei assessed in the ROI analysis above changed with rearing, using functional connectivity analysis (Perry et al., 2017; Opendak et al., 2019). This type of data representation provides an overview of patterns of change in the brain across broad networks of interconnected regions. Fos counts across individual animals for each brain region was used to construct bivariate correlation matrices for all pairwise combinations of brain regions analyzed for rearing condition. A single data point represents the correlation between Fos counts in a given brain region across all animals in a given condition with uptake in a different region in the same animals. Brain network matrices for the control-reared and LB maltreated-reared groups are shown in Figure 4C,D. Pairwise comparisons show several group differences: abused pups show higher connectivity between the BA-CeA (*p* = 0.015) and LA-BA (*p* = 0.043) than controls (Figure 4C, asterisks). In contrast, controls show higher connectivity between the IL-LA (*p* = 0.038) than abused adolescents. Finally, we observed that inter-connectivity between all amygdala subnuclei was higher in abused adolescents than controls (*p* = 0.027) and connectivity between the basolateral complex and other amygdala subnuclei was higher after abuse (Figure 4C,D, white rectangles; *p* = 0.004).

### Adolescent Correlational Analysis Between Amygdala-Freezing and vmPFC-Freezing

**FIGURE 5:**
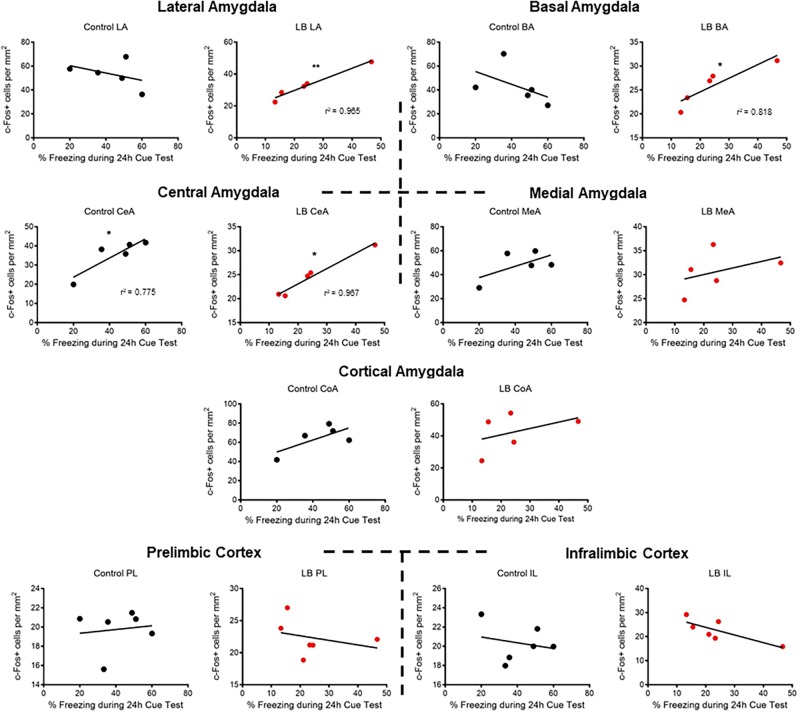
Correlations between cued threat response and ROI activation during adolescence. Correlations were computed between time spent freezing to CS and c-Fos levels for control-reared and maltreated (LB) pups. Pearson’s *r* values statistically different from 0 are. ^∗^*p* < 0.05, ^∗∗^*p* < 0.01.

Lastly, correlational analyses were performed between freezing behavior and amygdala-PFC ROIs in adolescent animals (Figure 5). In control-reared adolescent rats, we only observed a significant correlation between freezing levels and central amygdala c-Fos levels (*p* = 0.0487). In contrast, LB-reared pups showed significant correlations between freezing and activation of several amygdala subnuclei, including lateral, basal and central (LA: *p* = 0.003; BA: *p* = 0.039; CeA: *p* = 0.003).

## Discussion

Here, we showed that response to a learned threat increased from infancy through adolescence into adulthood, which was modified in a non-linear fashion by experience with LB maltreatment during early infancy. As summarized in Table 1, threat expression increased significantly across development, but this trajectory was altered by experience with infant maltreatment: relative to controls, freezing to a cue is increased in older maltreated infants (soon to be independent), which dips below control levels in adolescence before increasing above control levels in adulthood. The decreased response to the threat at adolescence may be due, at least in part, to reduced acquisition during conditioning. However, reduced response of the amygdala during cue testing may also contribute to adolescent reduced threat responding. To our knowledge, this is the first demonstration of these repeated dynamic developmental shifts on threat responding across development.

**Table 1:** Summary of the developmental neurobehavioral response to threat to a across development after experiencing infant trauma.

	PN18 late infancy	PN45 adolescence	>PN75 adults
Behavioral response to threat	Increased	Decreased	Increased
	**Integrating our adolescent neural results with existing literature**		
Amygdala	Increased	Decreased	Increased
PFC PL	NA	Increased	Increased


*Infants experienced PN8-12 maternal maltreatment with Scarcity-Adversity LB Model induced by providing mothers with insufficient bedding for nest building. Summarizing the behavioral data presented here, maltreatment produced a decrease response to threat while this same procedure produced a development switch to a maltreatment induced decrease followed by a developmental switch to adult increase. Next we integrate our adolescent amygdala and PL results with the existing literature on infants (Bath, 2018; Santiago et al., 2018; Opendak et al., 2019) and extensive adult literature (Barbosa Neto et al., 2012; Dannlowski et al., 2013; Jovanovic et al., 2014; Hartley and Lee, 2015; van Rooij et al., 2017; Demers et al., 2018; Norrholm and Jovanovic, 2018). Overall, the amygdala activity tracks trauma-induced changes in threat expression across the lifespan, while the PL does not.*

As noted in Table 1, the literature has already documented that amygdala-dependent learning occurs in late infancy, adolescence and adulthood (Pare et al., 2004; Pattwell et al., 2012; Hartley and Lee, 2015). The literature also documents its protracted development: amygdala dependent cue learning developmentally emerges at PN10 (Sullivan et al., 2000) and is functional in PN18 pups studied here (Opendak et al., 2018, 2019). Our lab has shown that early life maltreatment causes precocious incorporation of the amygdala into the threat learning circuit by a few days (PN7 rather than PN10) and the amygdala is hyperactive within a threat conditioning paradigm at PN18 to support enhanced learning shown in our previous rodent work (Moriceau et al., 2009a; Opendak et al., 2019).

At adolescence, following early infancy LB maltreatment, decreased responses to threat were accompanied by decreased c-Fos amygdala activation compared to controls across amygdala nuclei. This suggests widespread disruption of amygdala function. Specifically, the lower LB c-Fos in the cortical nucleus, considered the olfactory input area of the amygdala (Iurilli and Datta, 2017), suggests altered processing of the threat odor. The LB rats also exhibited lower lateral and basal nuclei c-Fos levels: this area is considered key for CS and US convergence and plasticity, suggesting learned association between the odor and the shock was also compromised (Romanski et al., 1993; Fanselow and LeDoux, 1999). Finally, the LB rats’ lower central nucleus (CeA) c-Fos suggests output is impaired (McKernan and Shinnick-Gallagher, 1997; Rogan et al., 1997; Phelps and LeDoux, 2005; Wilensky et al., 2006; Sah et al., 2008).

Using correlational analysis between freezing levels and amygdala nuclei (Figure 5), we observed that typically reared adolescents’ freezing was only correlated with the amygdala central nucleus, a brain area typically associated with output (LeDoux et al., 1988; LaLumiere, 2014). However, maltreated adolescents exhibited widespread correlations between freezing levels and specific amygdala nuclei, including the central amygdala and basolateral complex. Thus, while LB adolescent rats had lower c-Fos activation than controls, LB amygdala nuclei appeared to converge with the freezing response during the cue test.

Further analysis with functional connectivity between brain regions suggests that, within the amygdala, subnuclei exhibited enhanced communication following LB maltreatment, suggesting an expanded, diffuse response of the amygdala to a threat. Functional connectivity analysis within maltreated adolescents also suggests a switch from positive to negative connectivity between the amygdala-PFC, most notably between the IL and lateral amygdala. A similar pattern of transitioning positive to negative connectivity between the PFC and amygdala has been observed in developing children (Gee et al., 2013a). Furthermore, these patterns of altered interconnectivity are reminiscent of patterns observed in humans exhibiting pathology following early trauma (Yu et al., 2019).

LB maltreated adolescents exhibited enhanced PFC PL c-Fos expression, while IL levels were similar to controls. We interpret these results within the context of PFC function as a regulator of fear behavior by modulation of the amygdala and other brain areas (Pare et al., 2004). The vmPFC subdivisions IL and PL generally appear to have opposing effects on fear in adult animals: the PL promotes the expression of fear, and the IL inhibits it (Vidal-Gonzalez et al., 2006; Corcoran and Quirk, 2007). During adolescence, inactivation of the PL but not the IL was found to have an effect on freezing behavior during a predator odor (Chan et al., 2011). While we found enhanced PL activation in maltreated animals compared to controls, no correlation was found between freezing and IL or PL c-Fos activity for either typically reared and maltreated reared adolescents. In humans, the engagement of the vmPFC (PL and IL are not typically individually assessed in human fMRI) during threat and its enhanced function and functional connectivity has been seen in clinical populations (Phelps et al., 2004; Gee et al., 2013b; Birn et al., 2014; Schubert et al., 2015; Fonzo et al., 2016; Demers et al., 2018; Goetschius et al., 2019).

The literature also indicates that the PFC is functional during adolescent threat conditioning, although PFC development has been suggested to accelerate following early life stress (Yap and Richardson, 2005; Kim et al., 2009; Callaghan and Richardson, 2012; Li et al., 2012; Cowan et al., 2013). Specifically, in rat pups, the PFC does not seem to be functionally incorporated into the rat threat circuit until around PN23 (around weaning age) for innate threat responses (Chan et al., 2011; Takahashi, 2014) and learning about threat in a threat conditioning paradigm, behavioral expression, and during extinction in both humans and animal models (Nair et al., 2001; Kim et al., 2009; Ball and Slane, 2012; Li et al., 2012; Shechner et al., 2014; Almada et al., 2015; Heroux et al., 2017), as well as in other learning paradigms potentially at an earlier age (Lilliquist et al., 1999; Nair et al., 2001). It should be noted that the rodent and human PFC continues to develop though adolescence and appears to support unique features of adolescent extinction (Nair et al., 2001; Kim et al., 2009; Jovanovic et al., 2014; Lu et al., 2019).

## Conclusion

Our results suggest that experiencing maltreatment in early life sets in motion a complex developmental trajectory of neurobehavioral responses to threat that transitions across infancy, adolescence and into adulthood. These results complement research on children, where caregiver maltreatment is well-known to alter the threat system throughout development. Indeed, it is possible that this unique form of trauma in which the infant’s safety signal or “safe haven” (the caregiver) is actually the source of the threat (Kerns et al., 2015; Hornstein et al., 2016) produces distinct outcomes. Understanding unique trajectories following this specific developmental perturbation associated with maltreatment from the caregiver can help inform development of age-appropriate and trauma-specific treatments and interventions.

## Data Availability

The datasets generated for this study are available on request to the corresponding author.

## Ethics Statement

Procedures were approved by the Institutional Animal Care and Use Committee and followed National Institutes of Health guidelines.

## Author Contributions

AJ and RS designed and conducted the research. AJ, MO, and RS analyzed the data and wrote the manuscript. JL contributed to the data interpretation.

## Conflict of Interest Statement

The authors declare that the research was conducted in the absence of any commercial or financial relationships that could be construed as a potential conflict of interest.
